# Rehabilitation of a patient with amelogenesis imperfecta and severe open bite: A multidisciplinary approach

**DOI:** 10.1002/ccr3.1966

**Published:** 2018-12-19

**Authors:** Mahnaz Arshad, Gholamreza Shirani, Hossein‐Ali Mahgoli, Nastaran Vaziri

**Affiliations:** ^1^ Prosthodontics, Dental Research Center, Dentistry research institute, School of dentistry, International Campus Tehran University of Medical Sciences Tehran Iran; ^2^ Department of Oral & Maxillofacial Surgery, School of Dentistry Tehran University of Medical Sciences Tehran Iran; ^3^ International Campus Tehran University of Medical Sciences Tehran Iran

**Keywords:** adhesive cementation, amelogenesis imperfecta, full‐coverage indirect zirconia restoration

## Abstract

Amelogenesis imperfecta (AI) is typically associated with anterior open bite and a number of other dental problems, which require complex treatments such as orthognathic surgery. This case report describes management of a patient with AI and severe open bite via a multidisciplinary approach.

## 1 INTRODUCTION

Amelogenesis imperfecta (AI) is a rare inherited disorder characterized by incomplete formation or calcification of the enamel, affecting both the primary and permanent dentition.^1^, ^2^, ^3^ The global prevalence of AI is estimated between 1:1700 and 1:14 000, based on the study populations and the diagnostic criteria.^4^, ^5^, ^6^

Several types of AI exist based on clinical, radiographic, genetic, and histological findings. The most widely accepted classification of AI includes four main types: hypoplastic (type I), hypomaturation (type II), hypocalcification (type III), and hypomaturation‐hypoplasia with taurodontism (type IV).^1^, ^2^, ^3^

Impaired esthetics, tooth hypersensitivity, wide pulp chamber, higher risk of dental caries, and decreased occlusal vertical dimension are among the challenges faced in the management of AI. Dental anomalies associated with AI include enamel disorders, anterior open bite, pulpal calcifications, delayed eruption of teeth, pathological root and crown resorption, and taurodontism.^7^, ^8^

Management of AI requires a comprehensive treatment plan and a multidisciplinary approach involving a pediatric dentist, an orthodontist, a maxillofacial surgeon, and a prosthodontist; the latter plays a key role in coordinating all aspects of the treatment plan.^9^, ^10^, ^11^, ^12^ The four main treatment options available to correct anterior open bite include surgery, prosthetic crowns, composite or ceramic veneers (for cases with mild open bite), and veneering or crown placement combined with surgery; the latter option is often considered as an ideal treatment for anterior open bite.^5^

Herein, we report management of a rare case of severe hypomaturation‐hypoplasia with taurodontism (type IV AI) with class III malocclusion and severe anterior open bite via a multidisciplinary approach.

## 2 CASE REPORT

In 2013, a 27‐year‐old male patient presented to the Prosthodontics Department of Tehran University of Medical Sciences and complained of unattractive smile and difficult mastication.

His dental history revealed an unsuccessful maxillary Le Fort I orthognathic surgery in 2010 as an attempt to modify his class III malocclusion and correct his open bite, which relapsed afterward.

The patient, also suffering from mouth breathing, had a long oval face with a convex profile, incompetent lips with a nasolabial angle of 110°, and chin deficiency. The open bite and maximum mouth opening measured 10 and 48 mm, respectively.

### 2.1 Examination and diagnosis

**Figure 1:**
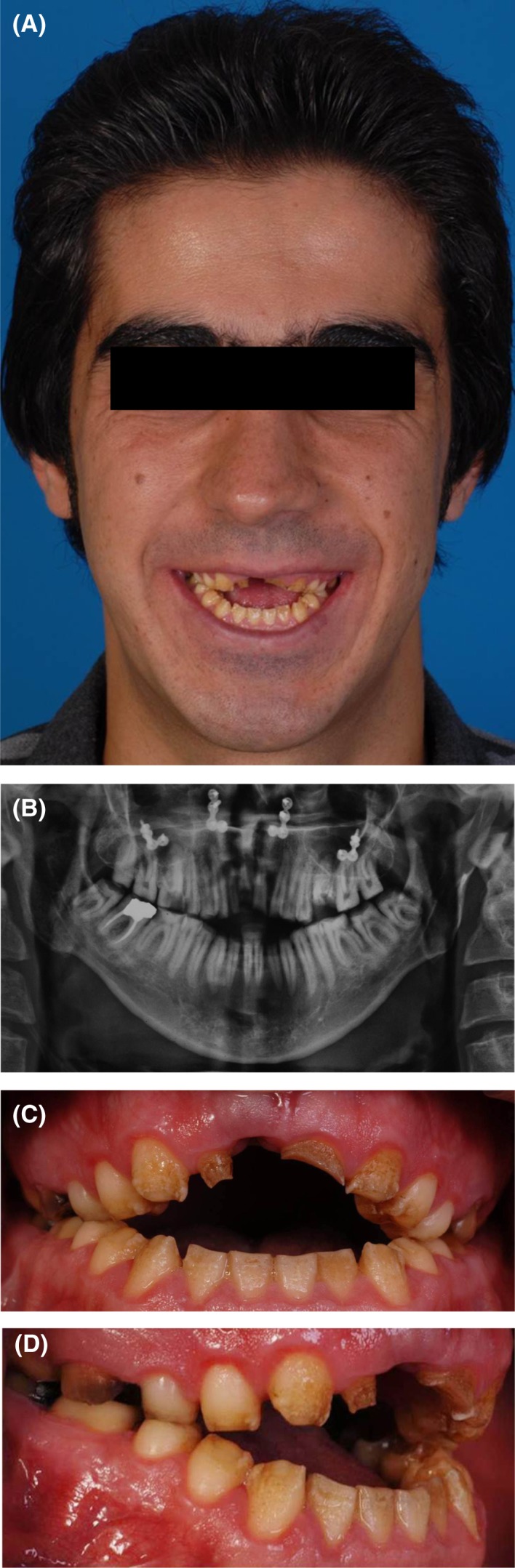
A, Profile view; B, Panoramic view; C, Intraoral frontal view; D, Intraoral right lateral view

Intraoral examinations revealed an ovoid arch form, deep palate, dental caries, short yellow‐brown pitted and porous teeth, hyperplastic and edematous gingiva (Figure 1), wide occlusal surfaces, and a buccolingual alveolar defect at the site of upper right central incisor. Other problems observed included tapered crowns, posterior occlusal contact to the second premolar, a biplanar open bite, reverse curve of Spee, no anterior guidance, low crown height of the posterior teeth, and no proximal contact. The upper left lateral incisor was missing and the upper right lateral incisor was peg‐shaped. The posterior teeth had wide pulp chambers and furcation proximity to the alveolar ridge, rendering crown lengthening surgery impossible. The diagnosis of hypomaturation‐hypoplasia with taurodontism (type IV AI) was made.

**Figure 2:**
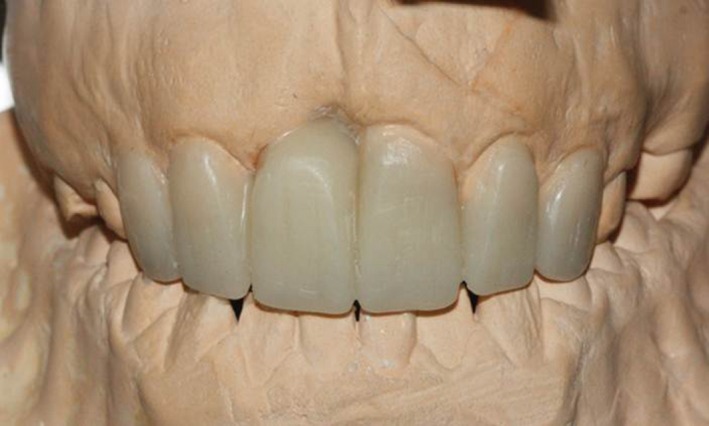
Diagnostic wax‐up for the analysis of the final crown‐root ratio needed to close the open bite by prosthetic crown placement

A crown‐root ratio analysis and a diagnostic wax‐up were carried out, which revealed that the open bite could not be corrected solely by prosthetic treatment (Figure 2). Further oral examinations revealed bleeding on probing, moderate to severe gingivitis, and moderate staining of teeth. Calculus was observed on the lingual surface of the mandibular anterior teeth, and the plaque index was calculated to be 80%. Thus, scaling and root planing was performed and an ointment was applied to prevent air contact with soft gingival tissue during mouth breathing while asleep. Oral hygiene instructions were also provided to the patient. As a result, his oral hygiene improved, his plaque index decreased to 30%, edema and inflammation were eliminated, and bleeding on probing stopped.

Diagnostic casts were poured and mounted on a Dentatus articulator (Dentatus USA Ltd., NY, USA) using a face bow. Lateral cephalometric studies and consultation with oral and maxillofacial surgeons led to the conclusion that the patient had maxillary deficiency, mandibular prognathism, and anterior open bite.

The patient had undergone Le Fort I osteotomy 8 years earlier with 4 mm advancement, 2 mm superior repositioning of the anterior maxilla, and 5 mm superior repositioning of the posterior maxilla, along with a mandibular setback surgery which was unsuccessful and relapsed.

### 2.2 Orthognathic surgery

According to the Epker and Dolphin imaging software prediction (Dolphin Imaging & Management Solutions, Chatsworth, CA, USA), the patient was scheduled for segmental orthognathic surgery to close the biplanar open bite. The first phase of the surgical procedure involved slight superior repositioning and setback of the anterior mandible, as a result of which, the curve of Spee, open bite, and class III jaw relationship improved.^13^ The second phase included a bilateral posterior maxillary segmental osteotomy for superior repositioning to correct the open bite, long face, and space shortage of the posterior teeth. Autogenous bone grafting was simultaneously performed at the peg lateral region for the anterior maxillary defect. Consequently, chin deficiency was slightly modified due to mandibular autorotation.

**Figure 3:**
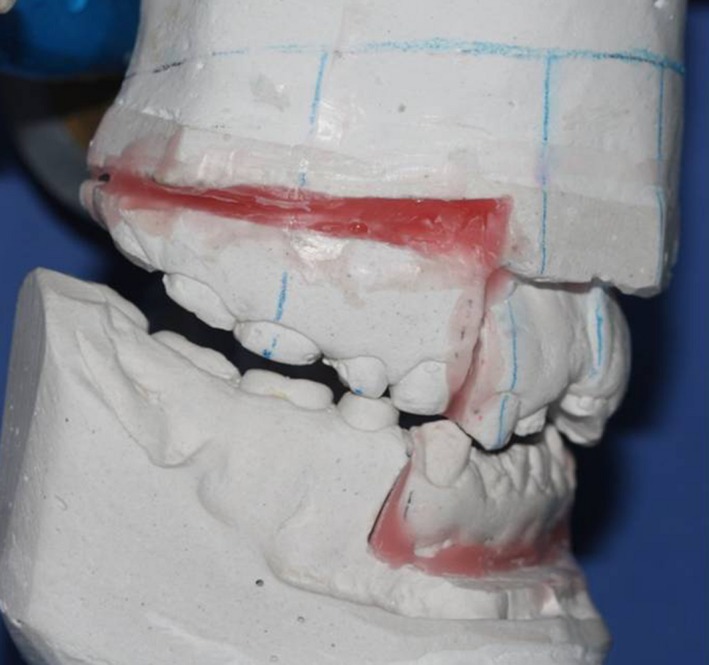
Model surgery

**Figure 4:**
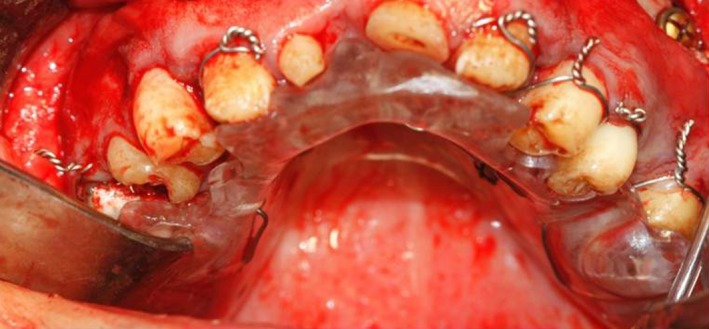
Stent fixation with wiring

Noticeably, in our patient, modifying the biplanar open bite with orthodontic treatment was not possible due to insufficient crown height and the enamel condition, which would not allow bracket bonding. To prevent toothache during the recovery period after orthognathic surgery, the following procedures were performed: The maxillary left first molar and the right central incisor were extracted. The maxillary right second premolar and molar, and the mandibular right first molar were endodontically treated, and the carious mandibular left first molar was restored with glass ionomer (GC Corporation, Tokyo, Japan). After conduction of a model surgery and fabrication of a surgical stent (Figure 3), the actual surgical procedure was carried out, and the open bite decreased by 7.4 mm. To prevent relapse and super‐eruption of teeth after surgery, a special space‐maintaining stent was fabricated to be fixed to the posterior maxillary segments since there was insufficient space for crowded teeth, due to short crowns of the posterior teeth and proximity of their furcation to the alveolar crest (Figure 4).

**Figure 5:**
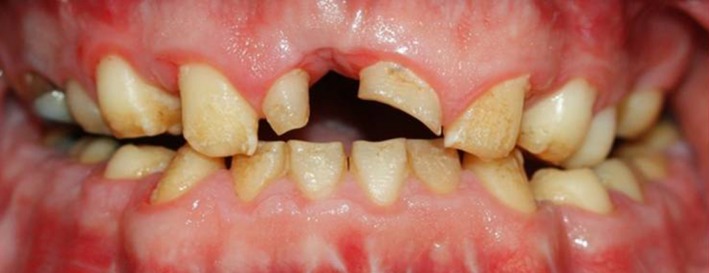
Post‐surgical intraoral frontal view

The upper right lateral incisor was not extracted during the orthognathic surgery, due to the possibility of fistulation of bone graft exposed to the oral cavity. Autogenous graft was harvested from the medial sinus wall and maxillary tuberosity and implanted in the upper right central incisor area. Postoperative results showed that a number of the patient's problems, namely the long face, chin strain, and open bite were successfully corrected (Figure 5), and an occlusal splint was fabricated for the patient to serve as a space maintainer.

**Figure 6:**
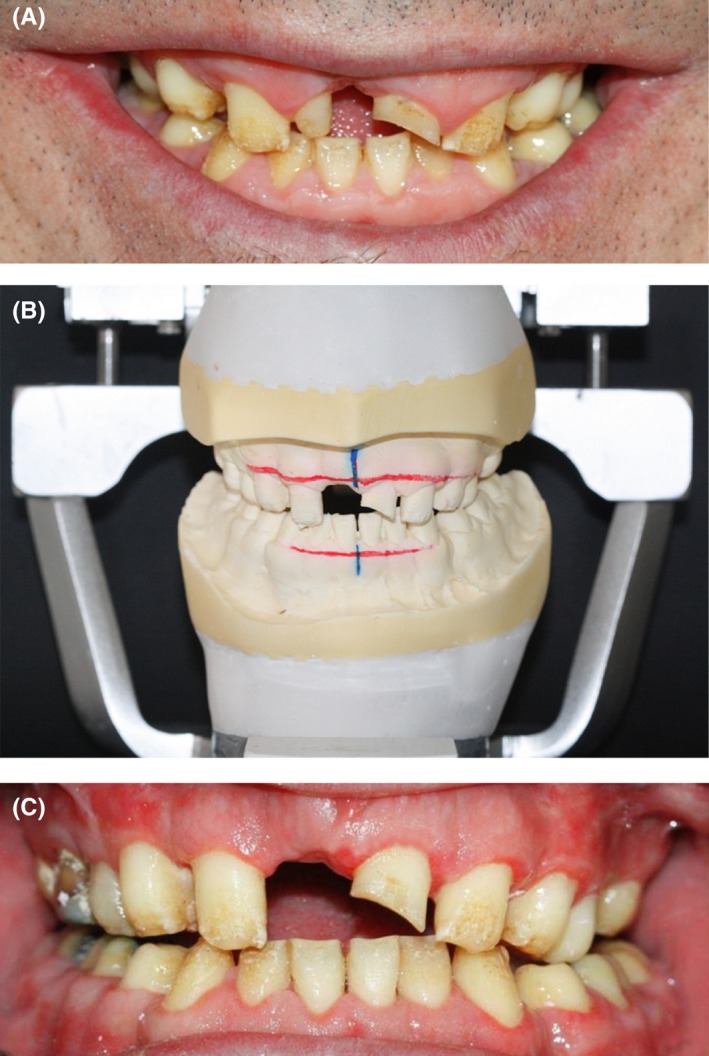
A, Smile line analysis; B, Smile line determination on the cast; C, Post‐crown lengthening: frontal view

After 2 months, the first impression was made with alginate (Zhermack, Badia Polesine, Italy) and a study cast was poured with dental stone (Kerr Dental, Orange County, CA, USA). Then, it was mounted on a Denar Mark II articulator (Whip Mix, Kentucky, USA). Esthetic analysis was carried out and the patient's smile line was transferred to the cast. Based on this cast, a surgical stent was fabricated for crown lengthening surgery and leveling of the gingiva (Figure 6A,B).

### 2.3 Periodontal surgery

It was decided to extract the peg lateral incisor due to its small crown‐root ratio, assessed in the diagnostic session, and anticipation of poor esthetics and lack of function in the future. The third molars were also extracted due to the proximity of their root furcation to the bone crest, which would complicate the treatment. After the extraction of lateral incisor, socket preservation was performed using demineralized bone matrix (Hamanand Saz Baft Kish Co., Kish, Iran) (Figure 6C). One month later, a primary impression was made. Another diagnostic cast was poured and mounted on a Denar Mark II articulator. Lip line, smile line, and midline were evaluated. The anterior plane of the mandible was analyzed, and the left central incisor, lateral incisor, and canine were reduced by 0.5, 1, and 1.5 mm, respectively.

**Figure 7:**
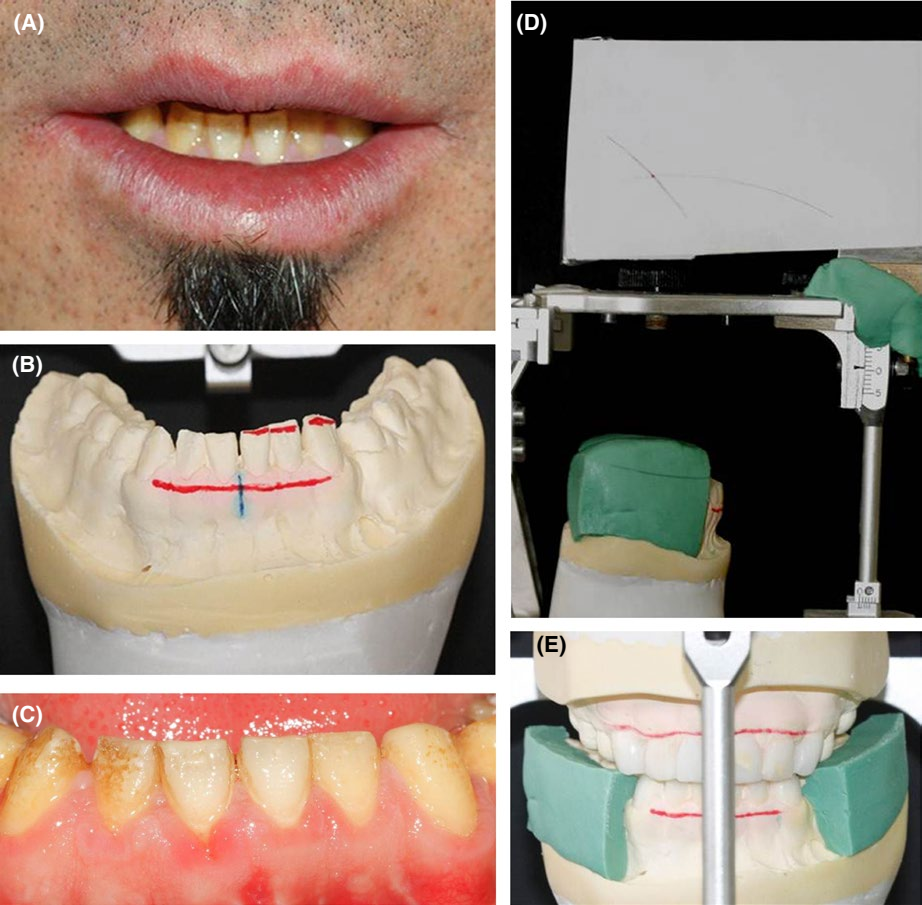
A, Mandibular anterior teeth analysis; B, Incisal edge reduction in the oral cavity; C, Incisal edge reduction on the cast; D, Broadrick flag; E, Occlusal plane determination with Broadrick flag

An anterior diagnostic wax‐up was fabricated and the occlusal plane was determined using the Broadrick flag. Based on the occlusal index, a complete wax‐up was performed (Figure 7).

### 2.4 Implantation phase

**Figure 8:**
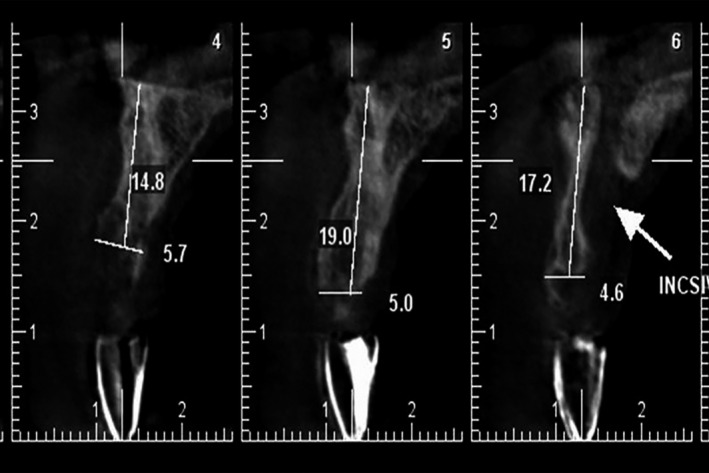
CBCT with radiographic stent

A radiographic barium sulfate coated stent was fabricated for cone‐beam computed tomography prior to dental implantation. The bone width and height and angulation of implant were evaluated. Dental implants (Xive, Dentsply Friadent, Mannheim, Germany) were then placed at the site of maxillary right central incisor, maxillary left second premolar, and mandibular left first molar (Figure 8). Closed sinus lift was also performed simultaneous with implant placement in the upper left premolar area.

**Figure 9:**
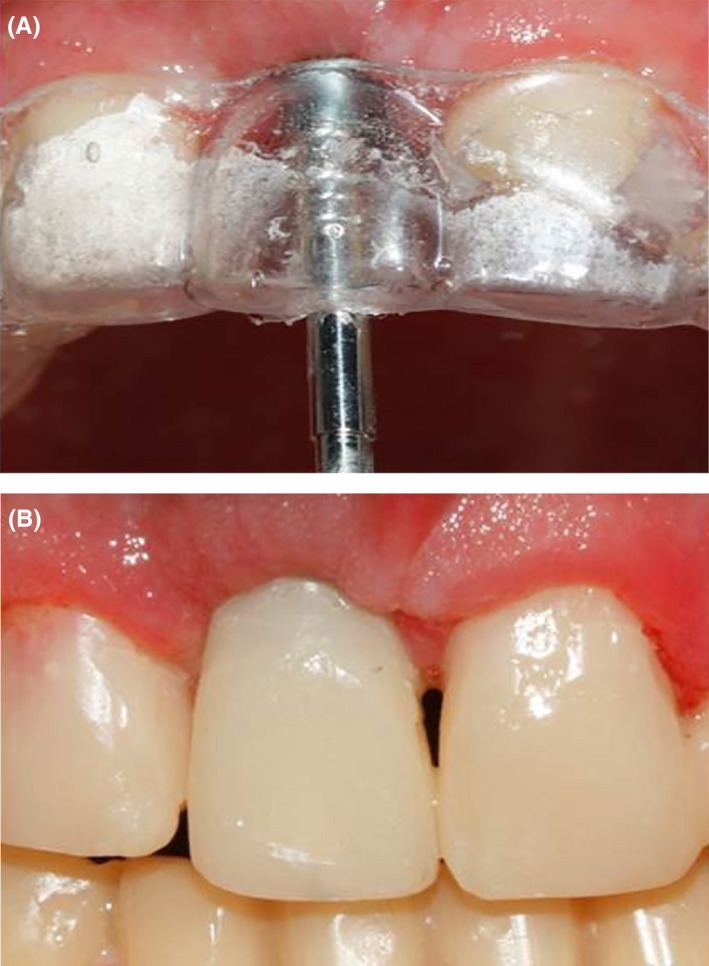
A, Fabrication of implant‐supported screw‐retained temporary crown; B, Final temporary crown

An immediate screw‐retained temporary crown was fabricated during the implant surgery session with a temporary abutment and a shell fabricated following wax‐up (Figure 9). The screw‐retained crown was used to form and prepare the soft tissue and dental papilla for the final impression.

### 2.5 Prosthetic phase

**Figure 10:**
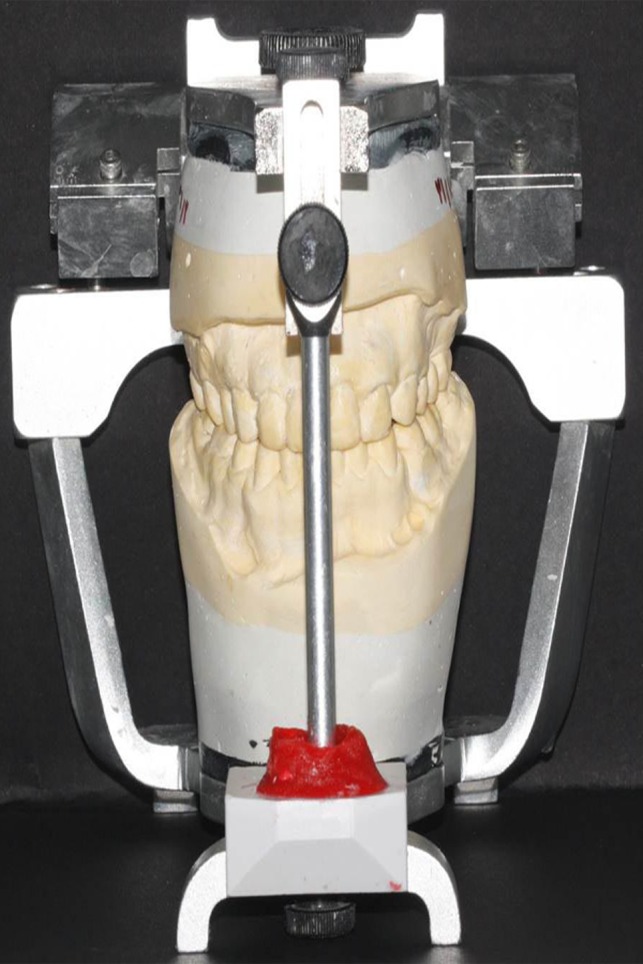
Fabrication of customized guiding table

The prosthetic phase began 2 months later. Due to the extensive loss of tooth structure, post patterns were fabricated for the upper right second premolar and first molar, and the lower right first and second molars.^14^ The posts were cast (Degubond, Dentsply Sirona Prosthetics, Pennsylvania, USA) and cemented with Panavia F 2.0 resin cement (Kuraray Co., Ltd., Tokyo, Japan). The final preparations for all‐ceramic crowns (butt joint margin) and laminate veneers (incisal overlap) were made for the six mandibular anterior teeth, and the teeth were restored with temporary crowns. A customized anterior guidance table and the posterior determinant of occlusion were designed based on the casts of the temporary crowns in Denar Mark II articulator, and the final crowns were fabricated accordingly (Figure 10).

The final impression was made using polyvinyl siloxane (Panasil, Kettenbach, Eschenburg, Germany) addition impression material. Subsequently, recording and mounting were performed. The master cast was poured, and cross‐recording and cross‐mounting were performed. Proper Esthetic Base abutment (Xive Dentsply Friadent, Mannheim, Germany) was chosen for the implants after obtaining a putty index and wax‐up. The dies were scanned by Cercon computer‐aided design/computer‐aided manufacturing system (Dentsply, sirona prosthetics, Pennsylvania, USA).

**Figure 11:**
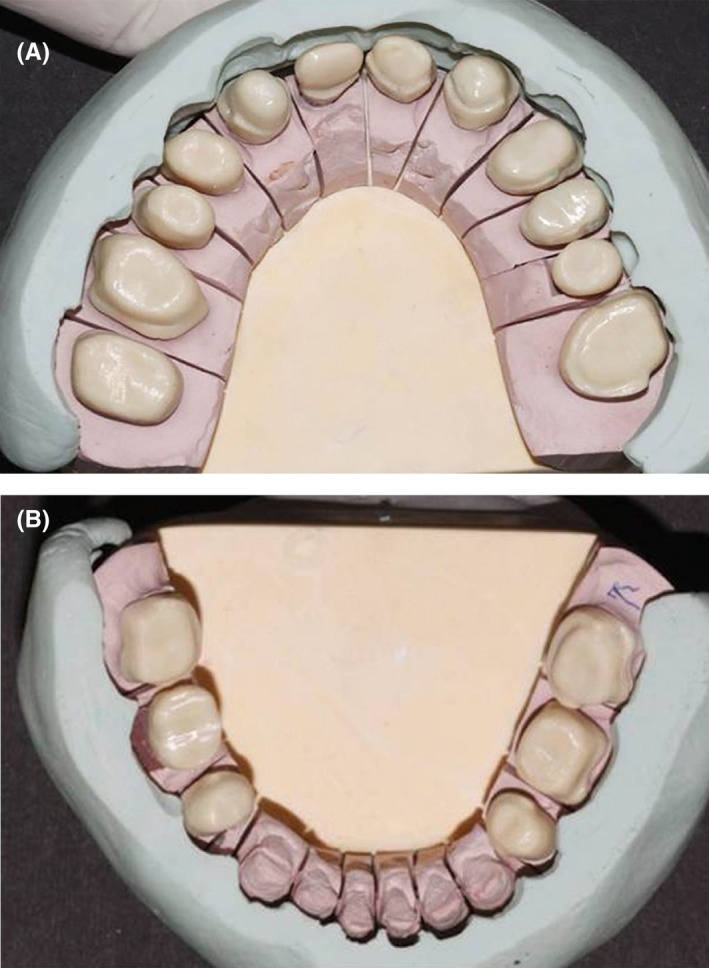
Framework analysis by comparison with the wax‐up putty index

To ensure optimal esthetics of the anterior restorations, zirconia frameworks (A2) were fabricated of ultra‐translucent multi‐layered and superior translucent multi‐layered ceramic (Porcelain Ceram Kiss/e.max: A2/A3, Katana Zirconia Block, Kuraray, Tokyo, Japan) (Figure 11). A radiograph was obtained to ensure correct seating of each framework. Porcelain was added to the framework and the crowns were tried in. Centric and eccentric occlusal contacts were then evaluated. The patient's occlusion was canine rise and mutually protected occlusion. During the delivery session, the crowns were cemented with temporary non‐eugenol cement (Kerr Dental). Choice 2 cement (Bisco, Illinois, USA), which is a light‐cure luting cement, was used for cementation of composite laminate veneers.

Oral hygiene instructions were given to the patient, and an occlusal guard was delivered to minimize the complications of zirconia‐based restorations.

**Figure 12:**
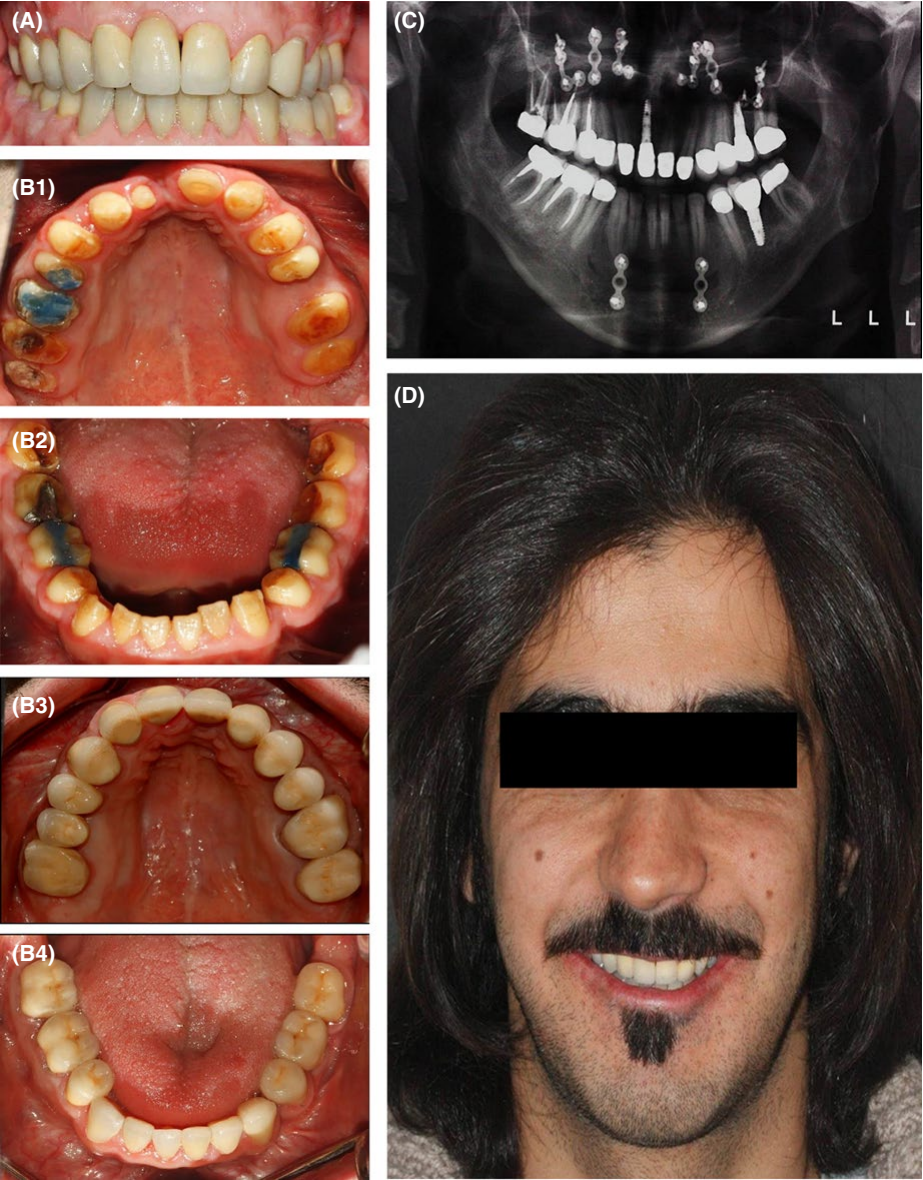
Final prosthesis. A, Intraoral frontal view; B1, Primary maxillary occlusal view; B2, Primary mandibular occlusal view; B3. Final maxillary occlusal view; B4, Final mandibular occlusal view; C, Panoramic view; D, Profile view

The patient was recalled 24 hours later for the first follow‐up session. He reported no complications and had no complaints. Two weeks later, permanent cementation was performed with Panavia F 2.0 (Kuraray, Tokyo, Japan) (Figure 12).^15^ An annual follow‐up was scheduled for the next 5 years with no reports of any complications or complaints. The patient was satisfied with the treatment outcome.

## 3 DISCUSSION

AI refers to a hereditary condition that affects the quality or quantity of the tooth enamel. It may involve both the primary and permanent dentition.^1^, ^2^, ^3^ AI is often associated with anterior open bite. Aside from problems in tooth eruption and the local factors that affect the growth and development of the dento‐alveolar system, impaired skeletal development can also be responsible for the anterior open bite usually associated with AI.^16^

Affected patients often experience impaired esthetics, tooth hypersensitivity, poor oral hygiene, higher risk of dental caries, excessive calculus formation, gingival hyperplasia, decreased vertical dimension due to the loss of tooth structure, and problems in mastication. Therefore, management of a patient with AI involves a complex interplay of various factors, which requires accurate diagnosis and treatment planning via a multidisciplinary approach. Participation of a pediatric dentist, an orthodontist, a maxillofacial surgeon, and a prosthodontist may be necessary in such multidisciplinary approaches.^7^, ^17^, ^18^, ^19^ To design an appropriate treatment plan, factors such as the quality and quantity of the enamel, age and socioeconomic status of the patient, the type and severity of the disorder, and the intraoral status should be taken into consideration.

In permanent dentition, the main treatment goals are to decrease tooth hypersensitivity, modify the vertical dimension of occlusion, and ensure satisfactory function and esthetics.^20^

Several approaches are available for correction of anterior open bite in AI including segmental orthognathic surgery, full‐coverage coronal restorations (crowns), porcelain laminate veneers, and composite laminate veneers.^9^ In our patient, two treatment methods were used simultaneously due to the severe anterior open bite and fracture of the anterior teeth namely segmental orthognathic surgery and application of crowns and composite laminate veneers.

The diagnostic wax‐up revealed that composite laminate veneers and crowns alone would not serve the purpose because the crowns would be too long and consequently unattractive, and that the crown‐root ratio would be inadequate, reducing the survival rate of the teeth. Therefore, the patient underwent segmental orthognatic surgery to partly close the anterior open bite, and then his anterior teeth were restored with composite laminate veneers (to prevent damage to the teeth) and the posterior teeth received stainless steel crowns. In patients with hypoplastic AI, enamel is deficient in quantity, but not in quality; therefore, it usually enables adhesive bonding, which means that composite resin restorations may be suitable to mask discolorations and improve the coronal morphology of such teeth.^21^

Bonding to the hypoplastic enamel is possible; but in our patient, we had to reduce the buccal surface of the anterior teeth by 0.5 mm to reach sound enamel for a strong bond to the enamel. After acid etching, laminate veneers along with an adhesive were used to restore the teeth.

The patient was followed up at 24 hours and 2 weeks, and annually thereafter for 5 years by clinical and radiographic examinations with no significant complications or complaints.

## 4 BENEFITS

Following consultation with dental professionals of different fields, an accurate multidisciplinary treatment plan was designed for our patient. Since his open bite had not been corrected and relapsed following his previous surgery in 2010, he was scheduled for segmental orthognathic surgery of the upper jaw following comprehensive assessments. Following prosthetic consultation, bone augmentation was performed simultaneous with implant placement. At the time of implant placement, gingival and soft tissue corrections were also performed. Finally, the prosthetic treatment phase was successfully accomplished. It is noteworthy that the treatment plan was designed taking into account the ideal esthetic and functional outcomes. Each phase was accurately implemented to achieve the ideal outcome.

## 5 CONCLUSION

This study described implementation of a multidisciplinary treatment plan designed for management of a patient suffering from hypoplastic AI with severe anterior open bite and class III malocclusion. In this case, the analysis of root‐crown ratio demonstrated that the open bite could not be corrected by prosthetic treatment alone. Therefore, a multidisciplinary treatment plan was designed following consultation with several dental professionals of different fields including a pediatric dentist, an orthodontist, a maxillofacial surgeon, and a prosthodontist. Then, the treatment plan was implemented step by step until successful results were achieved.

## CONFLICT OF INTEREST

The materials used in this case report are mentioned for clinical study purposes only. The authors do not have any financial interest in the companies whose materials are included in this article.

## AUTHOR CONTRIBUTION

AM: coordinated and carried out treatment, summarized case information, and drafted the manuscript. GS: carried out the treatment, participated in the follow‐up of the patient. MH: participated in patient care and coordinated treatment. NV: carried out the treatment, participated in the follow‐up of the patient, and drafted the manuscript. All authors approved the final manuscript.
